# Inspiring nurses’ sustainability mindset: Exploring the Mediating Role of Organizational Culture on the relationship between Pro-social Leader behaviors and nurses’ sustainability consciousness

**DOI:** 10.1186/s12912-024-02314-z

**Published:** 2024-09-26

**Authors:** Amal Diab Ghanem Atalla, Wafaa Hassan Mostafa, Mohamed Saad Saleh Ali

**Affiliations:** 1 Department of Nursing Administration, Faculty of Nursing, Alexandria University, Alexandria, Egypt; 2 Department of Nursing Administration, Faculty of Nursing, Damanhour University, El-Behira, Egypt

**Keywords:** Organizational culture, Pro-social leader behaviors, Nurses, Sustainability consciousness, Mediator

## Abstract

**Background:**

Since nurses are at the frontline of healthcare delivery, their actions and understanding of the environment have a big impact on how long healthcare systems can last. It is essential to comprehend the elements that impact nurses’ sustainability consciousness to encourage ecologically conscious actions in the healthcare industry.

**Aim:**

This study aimed to explore the relationship between pro-social leader behaviors and nurses’ sustainability consciousness and testify to the mediating role of organizational culture in this relationship.

**Design:**

A cross-sectional descriptive correlational design by STROBE criteria was used.

**Methods and tools:**

An approach to a judgmental non-probability sampling technique was employed to obtain data from 350 nurses in an Egyptian hospital. Three measurement surveys were employed: Organizational Culture Survey, Prosociality Scale, and, Sustainability Consciousness Questionnaire (SCQ-S). Relationships were shown using structural equation modeling and descriptive and inferential statistics.

**Results:**

53.4% of nurses have high perceptions of organizational culture, and the majority of nurses (85.7%) have high perceptions of prosocial leader behaviors. Furthermore, 60.9% of nurses have high perceptions of sustainability consciousness. Additionally, Prosocial leader behaviors positively correlated with organizational culture (*r* = 0.129) and nurses’ sustainability consciousness (*r* = 0.274). The indirect effect of prosocial leader behaviors on nurses’ sustainability consciousness through organizational culture is calculated by multiplying the coefficients of both direct effects (0.129 * 0.159 = 0.811). This means that for each unit increase in prosocial leader behaviors, we would expect a 0.811 unit increase in nurses’ sustainability consciousness through the mediating effect of organizational culture. The model appears to match the data well based on the model fit parameters (CFI = 1.000, IFI = 1.000, RMSEA = 0.114).

**Conclusions:**

The study highlights the impact of pro-social leader behaviors on nurses’ sustainability consciousness through the organizational culture as a mediating factor. **Nursing Implications**: Findings from this research can promote environmental stewardship and sustainable practices in the healthcare sector by illuminating the elements that can encourage and support a sustainability-oriented mindset among nurses. To promote a more sustainable future for the nursing profession, the findings can guide activities in nursing education, corporate culture transformation, and leadership development.

## Background

For healthcare organizations, leadership has become crucial, and a leader is required to set an example for others of positive social behaviors for nurses [1]. Pro-social behaviors focus on “giving something up” for the benefit of others without any personal benefit [2].

A roadmap for developing midwifery and nursing globally can be found in the World Health Organization’s “Global Strategic Directions for Nursing and Midwifery 2021–2025” publication. The purpose of this statement is to enhance access to healthcare worldwide by promoting the advancement of the nursing profession, actively influencing global health policy, and increasing competency development [3]. Global health policy is significantly influenced by the World Health Organization’s “State of the World’s Nursing 2020” report, which advocates for support for nursing leadership, employment, and education. By supporting fundamental competencies, solidifying the nursing profession, and forming nursing care paradigms, this study seeks to develop nursing and improve healthcare access on a worldwide scale [4].

The World Health Organization’s “Nursing and Midwifery” (2022) resource demonstrates the active involvement of nurses in the formulation of global health policy. By encouraging the development of important nursing and midwifery skills, supporting the advancement of these professions, and influencing the design of nursing care models, the ultimate purpose of this statement is to improve healthcare access and outcomes internationally [5]. Furthermore, the “Recover to Rebuild: Investing in the Nursing Workforce for Health System Effectiveness” (2023) report from the International Council of Nurses emphasizes the critical role that nurses play in strengthening and reconstructing healthcare systems. This report directly influences global health policy by arguing for increased funding for nursing education, employment, and leadership roles. Ultimately, it aims to improve healthcare outcomes and accessibility on a worldwide scale by promoting the advancement of the nursing profession and crucial nursing competency development [6].

## Literature review

### Pro-social leadership behaviors

Pro-social leadership behaviors are “a positive, effective influence, with constructive goals that serve the common good. Leaders are driven, and empathetic, and act to promote the welfare of people they have sworn to serve, regardless of the consequences or rewards they may get. Pro-social leadership behaviors include two main dimensions namely: pro-social actions and pro-social feelings. Pro-social behaviors come in many forms (e.g., sharing, caring, assisting, contributing, cooperating, assisting proactively, responding to requests for help, and protecting the organization) which stand for a broad behavioral dimension as opposed to the feeling dimension, or empathic feelings, which frequently drive other-oriented pro-social behaviors. Pro-social leadership behaviors concept in healthcare is becoming increasingly important because it contributes to staff and organizational development. Extra-role pro-social leader behaviors have been shown to improve social interactions’ reciprocity, cooperation, and solidarity, advance nurses’ well-being, and meet their needs by focusing on their career development, promoting nurses` growth and sustainability consciousness by sharing leaders’ views, establishing organization value, and encouraging mutual awareness [7].

### Nurses’ sustainability consciousness

The environmental, social, and economic facets of sustainability are integrated by nurses, highlighting the significance of sustainability knowledge, attitudes, and behaviors [8, 9]. Nurses’ awareness of ecological issues is measured by the environmental sustainability consciousness dimension. The long-term financial and personal welfare of nurses is a conscious concern in the economic dimension. The process of creating vibrant, long-lasting environments that promote wellness by understanding what people require from their lives and jobs is known as the social dimension. Furthermore, having a sustainable attitude means caring about sustainability, which leads to sustainable behaviors, but having a sustainable knowingness means being aware of the theoretical underpinnings of sustainable development. Behaviors that promote and aid in sustainable development are referred to as sustainability behaviors [10]. Educating nurses about sustainability can encourage them to implement sustainable practices in the clinical setting which decreases costs and wastes. Also, it plays an important role in organizational growth, financial sustainability, and competitive advantage; leading toward environmental protection, social welfare, and economic growth [11–13]. The pathway for the adoption of sustainability awareness leads via organizational culture where it is essential to building a sustainable, well-balanced workplace in today’s challenging contexts [14].

### Organizational culture

An organization’s management style and procedures are derived from its organizational culture, which is a collection of values and beliefs [15]. According to Quinn et al. (2020), an organization’s culture is a collective knowledge that employees recognize as what sets one organization apart from another. It influences behavior and the structure of management within the organization [16]. The four components of organizational culture are involvement, consistency, adaptation, and mission. Developing human capabilities at all levels, organizing around teams, and empowering employees are all examples of involvement. Consistency: refers to an organization’s well-coordinated and integrated activities and behavior that are based on a set of basic beliefs. Adaptability is the ability to take chances, learn from failures, and translate organizational environment expectations into action. It also refers to prior experience implementing change. A mission statement conveys the organization’s purpose and direction, identifies its aims and strategic objectives, and paints a picture of its future state [17]. Members are more cohesive and have a greater understanding of one another when they have the same culture. Organizational culture is essential to creating a safe healthcare system in the context of healthcare, hence it must exist [18].

### The underpinning of Batson’s theory

According to Batson’s theory, which maintains that empathy plays a significant part in forming a leader’s personality, prosocial leaders are driven by two ideals to act in ways that genuinely help society: empathy and altruism. When someone sees somebody in need, they have to respond to it or suppress their empathy. There are two parts to the ordinary leader development procedure. The term “expected representation” is the first item. It is an individual’s objectives, ideal future self, or sense of self. The construction of goals, the use of personal identity in the building of personal meaning, and the development of the moral self are all dependent on it. “Integration” is the second section. Through a dialectical process of thought, an individual compares their ultimate aims; such as being an agent with their current personal goals, which were established in reaction to empathy. the idealized appearance or moral persona they aspire to [19].

**Fig. 1:**
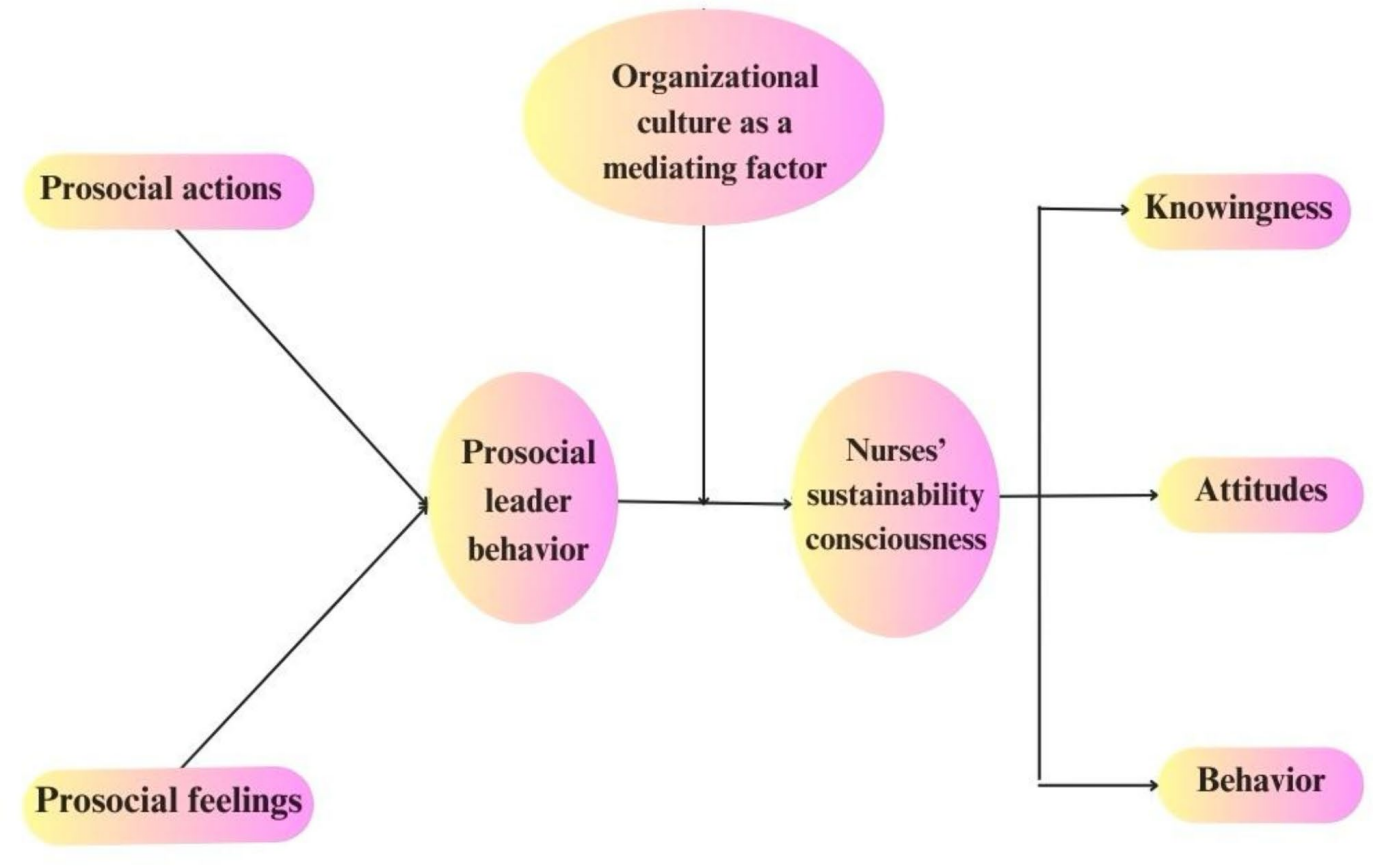
The researchers’ proposed conceptual framework of the study

From the aforementioned conceptualizations, we planned a conceptual model for this study (Fig. 1). Assumed that pro-social leader behavior is the independent variable, nurses’ sustainability consciousness is the dependent variable, and organizational culture acts as a mediating role, the following conceptual framework is postulated:

### Significance of the study

This study has significant implications for Egypt’s nursing workforce. It fills a significant void in the body of current literature. There is a paucity of research exploring the factors that influence the development of sustainability consciousness among nurses. This research fills this void by examining the intricate interactions between prosocial leadership behaviors, organizational culture, and nurses’ sustainability mindset.

Additionally, since nurses are at the forefront of healthcare delivery and have a critical role in putting sustainable practices into practice, this study contributes to promoting sustainable healthcare practices. The study’s understanding of the factors influencing nurses’ sustainability consciousness can help develop tactics that will enable them to take the lead in promoting environmental stewardship in hospital settings. The study on the mutually beneficial association between leader prosocial behaviors and sustainability consciousness in the success of nurses is immediately very important in the presence of Egyptian organizational culture. It offers insightful information on how to better the nation’s nursing profession and healthcare outcomes through modifying treatments, improving nursing practice, encouraging organizational development, and influencing legislative decisions.

Specifically, this study supports the larger global initiative to accomplish the Sustainable Development Goals (SDGs) of the United Nations, especially SDG 13 (Climate Action) and SDG 3 (Good Health and Well-Being). By promoting sustainable practices in nursing, the study contributes to the healthcare sector’s efforts to address environmental sustainability and social responsibility.

In inference, this study has numerous imperative theoretical implications as extending current leadership theories beyond typical performance-based outcomes by including pro-social leadership behaviors as antecedents of sustainability-related outcomes, such as nurses’ sustainability consciousness. This is in line with requests to investigate how leadership contributes to sustainability. Also, Analyzing organizational culture as a mediating mechanism brings to light how contextual elements shape how leadership influences outcomes connected to sustainability. This lends credence to the idea that the organizational environment influences how effective a leader’s actions are. Integrating sustainability-related concepts into the literature on organizational behavior involves positioning nurses’ consciousness of sustainability as a crucial outcome variable. This facilitates the communication between conventional organizational phenomena and sustainability. The suggested model takes a multilevel approach by considering the relationships between individual sustainability consciousness, company culture, and leader actions. This is in line with requests for additional multidimensional research in the fields of sustainability and leadership.

## Methods

### Study design

As per STROBE principles, a cross-sectional descriptive correlational research design was chosen.

### Setting

The study was carried out in the Ministry of Health and Population (MOHP)-affiliated Itay El Baroud General Hospital, which has 220 beds in total capacity. This covered all inpatient medical and surgical care units as well as Intensive Care Units (ICUs) (*n* = 16). These included: (1) medical units (*n* = 8), which included obstetric, pediatric, neurosurgical, dialysis, orthopedic, and poison units; (2) surgical units (*n* = 3), which included general surgical (Male and Female) and operation units; and (3) ICUs (*n* = 5), which included general, neonatal, pediatric, coronary care, and emergency units. This hospital began taking significant action to meet the patient safety requirements set forth by the General Authority for Health Accreditation and Regulation (GAHAR).

### Sampling

This is a judgmental non-probability sampling technique and not all nurses are included then, those who fulfill the criteria only. (we opt to look at the total population that meets a certain set of conditions: inclusion criteria). A total of 350 nurses who had worked in the aforementioned units for at least a year comprised the study group to familiarize them with the hospital’s administrative policies, procedures, and rules. When the information was being obtained, the nurses also needed to be there. The following criteria were fulfilled by nurses who were selected to take part in the study: The following requirements had to be met by them: (1) they had to remain on the working unit for a minimum of a year, and (2) they had to be directly giving patients with nursing care. The Raosoft sample size calculator was used to determine the appropriate sample size for a population of 660 people, with a 5-unit margin of error and a significance level of *p* ≤ 0.05. The recommended minimum sample size was calculated to be 244, and to ensure an adequate sample, 400 nurses were surveyed, with 350 nurses completing and returning the questionnaire, which met the target sample size.

### Ethical considerations

The research protocol was authorized by the Damanhour University Research Ethics Committee, which is a division of the College of Nursing. Nurses were informed of the aim of the study before providing their signed consent. To safeguard identity and secrecy, a code number was given to each questionnaire. The data was only used for research, as was assured to the nurses. The ability to leave the study has been confirmed.

### Study tools

**A structured questionnaire was employed to gather the data. The questionnaire comprised of four sections**,** as follows**:

#### Sociodemographic characteristics

The study participants’ years of service, years in the work unit, gender, age, education, and nursing experience were among the items the researchers questioned.

#### Organizational culture survey

Closed-ended surveys that were provided to the hospitals and tailored for staff were used to gather data. Items about organizational culture were included in the employee surveys, which were modified versions of Denison’s (1990) [20] organizational culture survey and validated by (Ashley & Brijball, 2024) [21]. Furthermore, nine measures are utilized to assess each of the four elements of corporate culture—involvement, consistency, flexibility, and mission—for a total of 36 questions. On a five-point Likert scale, respondents may select the option that most accurately expresses their viewpoint. A Likert scale, with numbers ranging from 1 (strongly disagree) to 5 (strongly agree), was employed in this investigation. Moreover, the Likert scale encompasses the entire spectrum of possible responses by ranking items based on agreement or disagreement. In addition, factor analysis was used to evaluate the validity of the questionnaire, and Cronbach’s coefficient alpha was used to evaluate its reliability. The findings demonstrate the extremely high degree of inter-item consistency reflected by the organizational culture questionnaire’s coefficient alpha (0.939), indicating the questionnaire’s reliability.

The Organizational Culture Survey has a mean score range of 36 to 180 overall. Cronbach’s alpha for the current investigation was 0.92. This was more significant than 0.35 and accounted for 82.151% of the variance in total. With a sampling adequacy of 0.923 according to Kaiser-Meyer-Olkin, the data was deemed appropriate for factor analysis. Furthermore, Bartlett’s test of sphericity achieved statistical significance (*P* = 0.000), confirming the correlation matrix’s factor capacity. As a result, the scale’s items were kept.

#### Prosociality scale

This scale was created and measures a variety of prosocial activities, such as sharing, lending a hand, and showing compassion, as well as sympathetic and empathetic responses. The scale compromised two main dimensions namely; prosocial actions (12 items) and prosocial feelings (4- items). Participants assessed their propensity to engage in prosocial behaviors (1 = never/nearly never true; 2 = occasionally true; 3 = sometimes true; 4 = often true; and 5 = almost always/always true) on a 16-item measure developed by Caprara et al. (2005a) [22] and validated by Luengo, et al., 2021) [23]. In the Caprara et al., (2005a) [22] investigation, the total Cronbach’s alpha for the scale was 0.94.

The total score falls between 16 and 80. The current study’s Cronbach alpha was 0.93. With a sampling adequacy of 0.923 according to Kaiser-Meyer-Olkin, the data was deemed appropriate for factor analysis. Furthermore, Bartlett’s test of sphericity achieved statistical significance (*P* = 0.000), confirming the correlation matrix’s factor capacity. As a result, the scale’s items were kept.

### Sustainability consciousness questionnaire (SCQ-S)

This questionnaire was developed by (Gericke, et al., 2019) [24], validated by (Bacci, et al., 2024) [25], and created in two versions, people’s attitudes, behavior, and knowledge of the environment, society, and economy can be assessed using the 49-item long version (SCQ-L) (nine valid and reliable subscales). The 27-item abbreviated form, or SCQ-S is useful for measuring Sustainability Consciousness. The scale exhibits outstanding psychometric quality in both versions. The results of analyses conducted with raw data and latent estimates, respectively, indicate a strong correlation between the short versions of all the measures, thus the short version was used in this study. The short version (SCQ-S) consists of three main dimensions: Knowingness, Attitudes, and Behaviour with three sub-dimensions in each main dimension. Three elements in each of the three sub-dimensions—environmental, social, and economic—make up a total of 27 items on the scale. The total score falls between 24 and 120. Cronbach’s alpha for the current investigation was 82. With a sample adequacy of 0.903, the Kaiser-Meyer-Olkin measure showed that the data could be used for factor analysis. Furthermore, Bartlett’s test of sphericity achieved statistical significance (*P* = 0.000), confirming the correlation matrix’s factor capacity. As a result, the scale’s items were kept.

### Pilot study

The pilot study was approved by 10% of the nurses (*n* = 35) to preserve the goods’ usefulness and simplicity and to spot any possible obstacles or problems during the data collection process. Nothing had to be changed. The main study did not involve any of the pilot study participants. The accuracy and inclusivity of the surveys were verified by the researchers.

### Data collection

To gather a sample list of all nurses and to get permission to meet them according to their schedules and break times, the researchers first visited the nurse managers of the units. After providing a thorough briefing and necessary instructions at the prearranged time, the researchers gave the questionnaires to each nurse who gave their agreement to participate in the study. Each nurse was given a two-minute explanation of the study’s goal before being asked to return it to the researcher. These scales were completed in front of the researcher to confirm the respondents’ objectivity, the coherence of their thoughts, and the completion of all questions. Completing the questions should take fifteen to twenty minutes. Three months passed between the first of January 2024 to the first of April 2024 to gather the data. All of the nurses’ questions were answered, and explanations were given.

### Data analysis

IBM SPSS AMOS (Version 23) and IBM SPSS Statistics (Version 23) were used to analyze the data. Frequency and percentage were used to describe the participant demographics. Means and standard deviations were used to describe the three primary study variables: pro-social leader behaviors, organizational culture, and nurses’ sustainability consciousness. Based on demographic features, changes in the research variable were found using an independent sample t-test and a one-way analysis of variance. The correlation between the key research variables was ascertained using Pearson’s correlation analysis. Regression models were used to ascertain the direct effect of pro-social leader behaviors on nurses’ sustainability consciousness. The indirect effect of pro-social leader behaviors on nurses’ sustainability consciousness as mediated by organizational culture was investigated using a structural equation model. To confirm the validity of the scale items, the study used composite reliability (CR) and Cronbach’s alpha. In addition, several confirmatory factor analyses were carried out to guarantee the accuracy of the study’s constituent parts.

## Results

Table 1 reveals that 83.7% of nurses are female, and 54.6% of nurses are from 40 to 50 years old. Furthermore, 78.0% of nurses are single. Moreover, 10%, 12.9%, and 37.1% are employed in internal medicine, surgery, and critical care units. 50.6% are technical nurses. The average number of years spent as a nurse is 7.76 ± 3.77. Additionally, 55.1% of nurses have worked in their hospital for 5 to 10 years.

**Table 1:** Distribution of the studied nurses according to demographic data (*n* = 350)

Demographic characteristics	No.	%
**Sex**		
Male	57	16.3
Female	293	83.7
**Age (years)**		
20–30	5	1.4
30–40	102	29.1
40–50	191	54.6
> 50	52	14.9
Mean ± SD	43.81 ± 6.96	
**Marital status**		
Single	273	78.0
Married	53	15.1
Divorced	5	1.4
Widowed	19	5.4
**Unit**		
Internal medicine	35	10
Surgical	45	12.9
Critical	130	37.1
Other	140	40
**Qualification**		
Professional	139	39.7
Technical	177	50.6
Practical	34	9.7
**Experience year of nursing**		
1–5	83	23.7
5–10	190	54.3
10–15	67	19.1
More than 15	10	2.9
Mean ± SD	7.76 ± 3.77	
**Experience hospital**		
1–5	80	22.9
5–10	193	55.1
10–15	67	19.1
More than 15	10	2.9
Mean ± SD	7.06 ± 3.75	

Table 2 clarifies that most nurses (53.4%) have high perceptions of organizational culture, and most nurses (85.7%) have high perceptions of prosocial leader behaviors. Furthermore, most nurses (60.9%) have high perceptions of sustainability consciousness. Finally, prosocial leader behaviors had the highest mean score (Mean ± SD = 80.56 ± 14.42).

**Table 2:** Distribution of the studied nurses according to their levels and mean percent score of Organizational Culture Instrument, Prosociality Scale Instrument, and Sustainability consciousness questionnaire (SCQ-S) (*n* = 350)

	Low (< 33.3%)		Moderate (33.3 – <66.6%)		High (≥ 66.6%)		Total score	Mean percent score	Mean score
	No.	%	No.	%	No.	%	Mean ± SD	Mean ± SD	Mean ± SD
A) Involvement	67	19.1	113	32.3	170	48.6	29.40 ± 9.15	56.67 ± 25.42	3.27 ± 1.02
B) Consistency	28	8.0	72	20.6	250	71.4	34.55 ± 6.88	70.96 ± 19.11	3.84 ± 0.76
C) Adaptability	10	2.9	154	44.0	186	53.1	31.37 ± 5.91	62.14 ± 16.41	3.49 ± 0.66
D) Mission	11	3.1	129	36.9	210	60.0	34.73 ± 7.13	71.47 ± 19.81	3.86 ± 0.79
**Overall Organizational Culture Instrument**	**13**	**3.7**	**150**	**42.9**	**187**	**53.4**	**130.05 ± 20.70**	**65.31 ± 14.38**	**3.61 ± 0.58**
A) Prosocial Actions	15	4.3	35	10.0	300	85.7	45.81 ± 8.29	79.12 ± 18.85	4.16 ± 0.75
B) Prosocial Feelings	0	0.0	30	8.6	320	91.4	17.12 ± 2.17	82.0 ± 13.59	4.28 ± 0.54
**Overall Prosociality Scale Instrument**	**4**	**1.1**	**46**	**13.1**	**300**	**85.7**	**62.93 ± 9.70**	**80.56 ± 14.42**	**4.22 ± 0.58**
I- Environmental	11	3.1	118	33.7	221	63.1	11.61 ± 2.57	71.76 ± 21.41	3.87 ± 0.86
II-Social	12	3.4	22	6.3	316	90.3	11.43 ± 1.59	70.24 ± 13.22	3.81 ± 0.53
III-Economic	14	4.0	127	36.3	209	59.7	11.53 ± 2.90	71.10 ± 24.20	3.84 ± 0.97
**A) Sustainability knowingness**	**13**	**3.7**	**55**	**15.7**	**282**	**80.6**	**34.57 ± 5.00**	**71.03 ± 13.89**	**3.84 ± 0.56**
I- Environmental	14	4.0	28	8.0	308	88.0	13.59 ± 2.61	88.29 ± 21.75	4.53 ± 0.87
II-Social	9	2.6	84	24.0	257	73.4	11.66 ± 2.28	72.14 ± 18.99	3.89 ± 0.76
III-Economic	25	7.1	196	56.0	129	36.9	10.07 ± 3.31	58.90 ± 27.59	3.36 ± 1.10
**B) Sustainability attitudes**	**11**	**3.1**	**103**	**29.4**	**236**	**67.4**	**35.32 ± 6.39**	**73.11 ± 17.75**	**3.92 ± 0.71**
I- Environmental	44	12.6	168	48.0	138	39.4	9.65 ± 2.94	55.40 ± 24.53	3.22 ± 0.98
II-Social	58	16.6	98	28.0	194	55.4	10.52 ± 3.34	62.67 ± 27.81	3.51 ± 1.11
III-Economic	80	22.9	78	22.3	192	54.9	10.91 ± 3.77	65.90 ± 31.39	3.64 ± 1.26
**C) Sustainability Behavior**	**26**	**7.4**	**198**	**56.6**	**126**	**36.0**	**31.08 ± 8.01**	**61.33 ± 22.24**	**3.45 ± 0.89**
**Overall Sustainability consciousness questionnaire**	**12**	**3.4**	**125**	**35.7**	**213**	**60.9**	**100.97 ± 14.21**	**68.49 ± 13.16**	**3.74 ± 0.53**

SD: Standard deviation

Table 3 provides a correlation matrix of the relationships between prosocial leader behaviors, organizational culture, and nurses’ sustainability consciousness based on a sample size of 350. All correlations were statistically significant at *p* = 0.05, according to the data. Prosocial leader behaviors positively correlated with organizational culture (*r* = 0.129) and nurses’ sustainability consciousness (*r* = 0.274). This suggests that improvements in prosocial leader behaviors are associated with improvements in organizational culture and nurses’ sustainability consciousness. Organizational culture, and nurses’ sustainability consciousness also positively correlated (*r* = 0.192), indicating that they tend to improve together.

**Table 3:** Correlation between the studied variables (*n* = 350)

		A) Involvement	B) Consistency	C) Adaptability	D) Mission	Overall Organizational Culture Instrument	A) Prosocial Actions	B) Prosocial Feelings	Overall Prosociality Scale Instrument	I- Environmental	II-Social	III-Economic	A) Sustainability knowingness	I- Environmental	II-Social	III-Economic	B) Sustainability attitudes	I- Environmental	II-Social	III-Economic	C) Sustainability Behavior	Overall Sustainability consciousness questionnaire
A) Involvement	**r**																					
	**p**																					
B) Consistency	**r**	0.297*																				
	**p**	< 0.001*																				
C) Adaptability	**r**	0.396*	0.247*																			
	**p**	< 0.001*	< 0.001*																			
D) Mission	**r**	0.101	0.755*	0.287*																		
	**p**	0.059	< 0.001*	< 0.001*																		
**Overall Organizational Culture Instrument**	**r**	0.689*	0.794*	0.642*	0.722*																	
	**p**	< 0.001*	< 0.001*	< 0.001*	< 0.001*																	
A) Prosocial Actions	**r**	0.019	0.100	0.056	0.072	0.083																
	**p**	0.722	0.062	0.294	0.177	0.123																
B) Prosocial Feelings	**r**	0.012	0.439*	0.047	0.312*	0.262*	0.570*															
	**p**	0.818	< 0.001*	0.377	< 0.001*	< 0.001*	< 0.001*															
**Overall Prosociality Scale Instrument**	**r**	0.014	0.184*	0.059	0.132*	0.129*	0.983*	0.711*														
	**p**	0.800	0.001*	0.274	0.013*	0.015*	< 0.001*	< 0.001*														
I- Environmental	**r**	0.099	0.432*	0.182*	0.505*	0.222*	0.080	0.323*	0.141*													
	**p**	0.065	< 0.001*	0.001*	< 0.001*	< 0.001*	0.135	< 0.001*	0.008*													
II-Social	**r**	0.037	0.002	0.031	0.069	0.015	0.187*	0.189*	0.202*	0.578*												
	**p**	0.488	0.965	0.567	0.195	0.774	< 0.001*	< 0.001*	< 0.001*	< 0.001*												
III-Economic	**r**	0.150*	0.238*	0.237*	0.298*	0.048	0.238*	0.183*	0.245*	0.176*	0.583*											
	**p**	0.005*	< 0.001*	< 0.001*	< 0.001*	0.375	< 0.001*	0.001*	< 0.001*	0.001*	< 0.001*											
**A) Sustainability knowingness**	**r**	0.024	0.083	0.054	0.108*	0.091	0.239*	0.332*	0.279*	0.595*	0.952*	0.675*										
	**p**	0.648	0.120	0.311	0.043*	0.088	< 0.001*	< 0.001*	< 0.001*	< 0.001*	< 0.001*	< 0.001*										
I- Environmental	**r**	0.060	0.003	0.014	0.094	0.003	0.173*	0.094	0.169*	0.461*	0.745*	0.487*	0.757*									
	**p**	0.261	0.961	0.798	0.078	0.957	0.001*	0.079	0.002*	< 0.001*	< 0.001*	< 0.001*	< 0.001*									
II-Social	**r**	0.094	0.253*	0.198*	0.129*	0.030	0.132*	0.231*	0.165*	0.472*	0.507*	0.133*	0.481*	0.347*								
	**p**	0.079	< 0.001*	< 0.001*	0.016*	0.574	0.013*	< 0.001*	0.002*	< 0.001*	< 0.001*	0.013*	< 0.001*	< 0.001*								
III-Economic	**r**	0.105*	0.385*	0.337*	0.358*	0.109*	0.013	0.119*	0.015	0.744*	0.392*	0.294*	0.336*	0.332*	0.531*							
	**p**	0.049*	< 0.001*	< 0.001*	< 0.001*	0.042*	0.804	0.026*	0.777	< 0.001*	< 0.001*	< 0.001*	< 0.001*	< 0.001*	< 0.001*							
**B) Sustainability attitudes**	**r**	0.063	0.289*	0.240*	0.193*	0.066	0.111*	0.182*	0.135*	0.742*	0.688*	0.094	0.655*	0.704*	0.773*	0.843*						
	**p**	0.236	< 0.001*	< 0.001*	< 0.001*	0.219	0.038*	0.001*	0.011*	< 0.001*	< 0.001*	0.078	< 0.001*	< 0.001*	< 0.001*	< 0.001*						
I- Environmental	**r**	0.032	0.072	0.053	0.102	0.060	0.122*	0.041	0.113*	0.036	0.035	0.143*	0.113*	0.133*	0.140*	0.069	0.031					
	**p**	0.545	0.178	0.320	0.058	0.264	0.023*	0.449	0.034*	0.506	0.509	0.007*	0.035*	0.013*	0.009*	0.196	0.557					
II-Social	**r**	0.286*	0.057	0.076	0.114*	0.128*	0.000	0.035	0.007	0.100	0.095	0.087	0.132*	0.301*	0.009	0.035	0.145*	0.638*				
	**p**	< 0.001*	0.289	0.154	0.033*	0.017*	0.993	0.513	0.890	0.061	0.077	0.105	0.014*	< 0.001*	0.869	0.509	0.007*	< 0.001*				
III-Economic	**r**	0.389*	0.433*	0.019	0.303*	0.426*	0.269*	0.539*	0.351*	0.315*	0.123*	0.041	0.225*	0.052	0.259*	0.145*	0.188*	0.364*	0.378*			
	**p**	< 0.001*	< 0.001*	0.723	< 0.001*	< 0.001*	< 0.001*	< 0.001*	< 0.001*	< 0.001*	0.022*	0.449	< 0.001*	0.333	< 0.001*	0.007*	< 0.001*	< 0.001*	< 0.001*			
**C) Sustainability Behavior**	**r**	0.314*	0.201*	0.021	0.058	0.231*	0.172*	0.254*	0.204*	0.203*	0.110*	0.108*	0.202*	0.199*	0.074	0.057	0.137*	0.805*	0.829*	0.762*		
	**p**	< 0.001*	< 0.001*	0.693	0.282	< 0.001*	0.001*	< 0.001*	< 0.001*	< 0.001*	0.039*	0.044*	< 0.001*	< 0.001*	0.166	0.285	0.010*	< 0.001*	< 0.001*	< 0.001*		
**Overall Sustainability consciousness questionnaire**	**r**	0.157*	0.272*	0.077	0.157*	0.192*	0.231*	0.342*	0.274*	0.658*	0.707*	0.341*	0.760*	0.695*	0.559*	0.530*	0.757*	0.479*	0.578*	0.593*	0.696*	
	**p**	0.003*	< 0.001*	0.152	0.003*	< 0.001*	< 0.001*	< 0.001*	< 0.001*	< 0.001*	< 0.001*	< 0.001*	< 0.001*	< 0.001*	< 0.001*	< 0.001*	< 0.001*	< 0.001*	< 0.001*	< 0.001*	< 0.001*	

r: Pearson coefficient *: Statistically significant at *p* ≤ 0.05

**Fig. 2:**
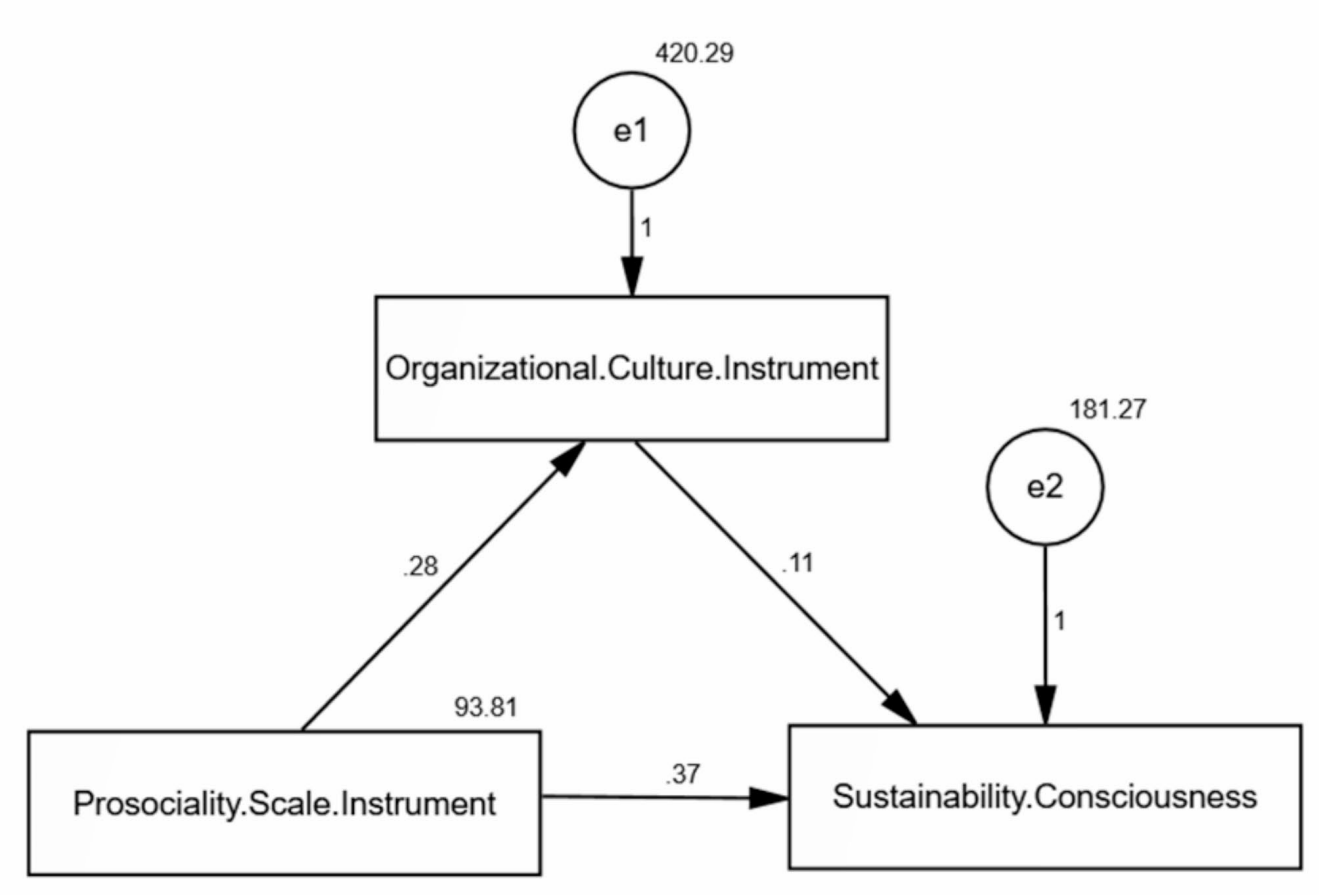
Path analysis of the direct and indirect effect of Prosociality Scale Instrument on Sustainability consciousness: Organizational Culture Instrument as a mediator

Table 4; Fig. 2 present the direct and indirect effects of prosocial leader behaviors, sustainability consciousness, and organizational culture. Prosocial leader behaviors (independent variable) directly affect organizational culture (mediator). This is represented by the path coefficient of 0.129 (p 0.015). Organizational culture (mediator) directly affects nurses’ sustainability consciousness (dependent variable). This is represented by the path coefficient of 0.159 (*p* = 0.002). Prosocial leader behaviors (independent variable) directly affect nurses’ sustainability consciousness (dependent variable). This is represented by the path coefficient of 0.253 (*p* < 0.001). The indirect effect of prosocial leader behaviors on nurses’ sustainability consciousness through organizational culture is calculated by multiplying the coefficients of both direct effects (0.129 * 0.159 = 0.811). This means that for each unit increase in prosocial leader behaviors, we would expect a 0.811 unit increase in nurses’ sustainability consciousness through the mediating effect of organizational culture. The model appears to match the data well based on the model fit parameters (CFI = 1.000, IFI = 1.000, RMSEA = 0.114). In terms of degrees of freedom, the Chi-square value (X2/df = 14.209/3) represents the difference between the model and the data. A better fit is indicated by a smaller value. There is a perfect fit when both the incremental fit index (IFI) and comparative fit index (CFI) equal 1.000. A fair approximation error is shown by the Root Mean Square Error of Approximation (RMSEA) of 0.114, which is less than the generally accepted criterion of.08. All of these indicators point to a good representation of the observed data by the model.

**Table 4:** The direct and indirect effect of Prosociality Scale Instrument on Sustainability consciousness: Organizational Culture Instrument as a mediator

			Direct effect	Indirect effect	Estimate	S.E.	C.*R*.	*P*
Organizational Culture Instrument	<---	Prosociality Scale Instrument	0.276	-	0.129	0.013	2.436*	0.015*
Sustainability consciousness	<---	Prosociality Scale Instrument	0.371	0.030	0.253	0.075	4.943*	< 0.001*
Sustainability consciousness	<---	Organizational Culture Instrument	0.109	-	0.159	0.035	3.112*	0.002*

Model fit parameters CFI; IFI; RMSEA (1.000; 1.000; 0.114).

Model χ^2^/df. 14.209/3 *p* ≤ 0.001.

CFI: Comparative Fit Index, IFI: Incremental Fit Index, RMSEA: Root Mean Square Error of Approximation

## Discussion

The idea of prosocial leadership behaviors is becoming more and more significant in the healthcare industry since it supports organizational and staff growth. By sharing leaders’ perspectives on the environment, developing organizational values, and fostering mutual awareness, extra-role prosocial leader behaviors have been demonstrated to improve nurses’ growth and sustainability consciousness [14, 26]. The process by which organizational culture adopts sustainability awareness, whereby becomes essential for managing and advancing sustainable growth in today’s demanding environments [11].

### Perceived level of pro-social leader behaviors, nurses’ sustainability consciousness and organizational culture among nurses

The study outcomes confirmed that most nurses perceived that their managers have a high level of prosocial leadership behaviors. This could be explained by the nurses’ perception that the managers serve as an example of positive social behaviors; they are upbeat, effective at influencing staff nurses, capable of enacting change, and focused on the needs of the larger group rather than their interests. They also protect the healthcare organization and offer proactive assistance in response to requests for assistance. The results of this study are consistent with those of (El-sawah and El-kholy, 2024) [7], (Mekawy, 2023) [27]_,_ (Luthufi, et al., 2021) [28] and (Feather, et al., 2018) [29], who reported that there was a high level of responsiveness from staff nurses to overall prosocial leadership.

This study also discovered that most nurses have high perceptions of sustainability consciousness. This might be attributed to that staff nurses are conscious of and concerned about sustainable development, which translates into sustainable development behaviors such as cutting back on water use in hospitals, learning how to safeguard hospitals from natural disasters, abiding by the rules and laws about the preservation of the hospital’s environment, using less packaging and disposable items, and managing waste. The findings of this study are in line with those of (El-sawah and El-kholy, 2024) [7], (Frostenson, et al. 2022) [30], (Afzal and Lim, 2022) [31], and (Elg, et al. 2021) [32], who discovered that the overall organizational sustainability levels were highly rated by staff nurses.

Furthermore, the study results revealed that most nurses have high perceptions of organizational culture. This could be explained by the notion that nurses possess several key attributes, including empowerment, teamwork, shared values, well-coordinated and integrated activities, risk-taking, learning from errors, and change-making experience. They also have a distinct direction and goal that includes a future vision for the company as well as organizational goals and strategic objectives. The present study’s results align with the findings of (Ashley & Brijball, 2024) [21] and (Budi & Abidin, 2021) [33], which indicated that nurses exhibited a high degree of involvement, consistency, adaptability, and mission within their organizational culture.

### Relationship among pro-social leader behaviors, nurses’ sustainability consciousness and organizational culture among nurses

The findings of this study revealed that nurses’ sustainability consciousness was correlated with prosocial leader behaviors. This finding implies that prosocial leader behavior is one of the leading indicators of nurses’ sustainability consciousness. This can be explained by the fact that prosocial leadership behaviors encourage nurses to be conscious of sustainability through exchanging perspectives on the healthcare environment, defining organizational values, and supporting sustainability consciousness as a means of ensuring patient safety and high-quality care. This finding is consistent with a study by (El-sawah & El-kholy, 2024) [7], (Nisar, et al., 2021) [34], and (Kim, et al., 2019) [12], who claimed that prosocial leadership and organizational sustainability had a statistically significant positive association.

Furthermore, the findings of this study displayed organizational culture as a mediator between prosocial leader behaviors and nurses’ sustainability consciousness. This outcome can be explained by the way that prosocial leaders have an impact on the attitudes and beliefs of staff nurses, which creates a positive organizational culture and raises staff nurses’ awareness of sustainability. This, in turn, encourages staff nurses to adopt sustainable practices in the healthcare setting, which reduces costs and waste and promotes social welfare, environmental protection, and economic growth. The study’s results are consistent with those of (El-sawah & El-kholy, 2024) [7], (Alvarez & Medina, 2023) [35], (Assoratgoon & Kantabutra, 2023) [36], and (Nisar, et al., 2021) [34], who revealed that prosocial leadership behaviors shape nurses’ attitudes and beliefs, creating a healthy organizational culture that raises nurses’ consciousness of sustainability.

### Standardized regression coefficient weights among pro-social leader behaviors, and nurses’ sustainability consciousness with the mediating role of organizational culture

Similarly, organizational culture and nurses’ sustainability consciousness were favorably correlated with prosocial leader behaviors. Organizational culture (mediator) directly affects nurses’ sustainability consciousness (dependent variable). The reason for this outcome could be that the adoption of sustainability consciousness in healthcare organizations is facilitated by organizational culture, which establishes shared values and attitudes among staff nurses on sustainability awareness and sustainable development in the face of today’s complex circumstances. This result is in line with the findings of (Macagnan & Seibert, 2022) [37], (Kantaburta, 2021) [38], and (Isensee, 2020) [39], who claimed that organizational culture positively correlated with nurses’ sustainability consciousness.

### Strengths and limitations

This study includes return estimations. Accurate information in case series and reports is less susceptible to biases because the cross-sectional methodology made it possible to evaluate multiple characteristics in the sample at once. The study extends our knowledge of the influence of pro-social leader behaviors on nurses’ sustainability consciousness, an issue that gets little attention in the healthcare sector by utilizing organizational culture as a mediating factor as viewed by nurses.

There are certain limitations, though. First, the nurses were selected through convenience sampling or from a single healthcare organization thus, the results may not be representative of the broader nursing population. Additionally, the study was conducted in a specific geographical area or setting, which could also limit the generalizability of the findings to nurses in different cultural or organizational contexts. There is a need for further research to validate the findings in more diverse samples and settings. Second, the only dependent variable in this study was the relationship between pro-social leader behaviors and nurses’ sustainability consciousness, with organizational culture serving as a mediating factor. Thirdly, the study’s unclear statement on the impact of control factors could make it more difficult to evaluate the findings. Subsequent research endeavors may evaluate supplementary factors that influence nurses’ awareness of sustainability. Also, the data entry and clearing process for the paper-based questionnaire was very labor-intensive. Finally, there is no evidence linking any of the study’s components to a causal relationship. After all, the goal was to investigate the relationship between the variables.

## Conclusion

This study adds to the expanding corpus of research on sustainability in healthcare, particularly in the nursing context It provides empirical evidence on the interplay between prosocial leadership behaviors, organizational culture, and nurses’ sustainability mindset, which can inform future research and practical applications. The human-centeredness and caring amenities of the nursing profession make it stand out. Only nurses adhere to certain values when interacting with patients and their families [40]. Healthcare leaders should recognize the interplay between leaders’ prosocial behaviors and organizational aspects such as organizational culture, and nurses’ sustainability mindset [41]. This means that fostering a culture of support and continual learning and skill development should be a top priority for healthcare companies. Hospital administrators should encourage organized networks of communication between nursing leaders and subordinates, encouraging nurses to participate in committee meetings and decision-making [42]. Overall, this study aims to shed light on the factors that can inspire and nurture a sustainability-oriented mindset among nurses, with the ultimate goal of promoting sustainable practices and environmental stewardship in the healthcare industry. The findings can inform leadership development, organizational culture change, and nursing education initiatives to foster a more sustainable future in the nursing profession.

The present study’s results suggest that pro-social behaviors, sustainability consciousness, and organizational culture are positively correlated. Specifically, there is a statistically significant positive correlation between pro-social behaviors and sustainability consciousness. Additionally, there is a statistically significant positive relationship between organizational culture and pro-social behavior. Additionally, a statistically significant positive association between organizational culture and sustainability consciousness. There is an impact of pro-social behaviors on sustainability consciousness through the organizational culture as a mediating factor.

### Implications for nursing practice, education and policy

The practice of nursing will be significantly impacted by these findings. They recommend that healthcare institutions give pro-social leadership behavior development top priority and cultivate an environment that supports and encourages nurses’ awareness of sustainability. This could result in several advantages, including enhanced environmental sustainability procedures in healthcare environments, which help lower waste, energy use, and carbon emissions. Increased involvement and job satisfaction among nurses because they feel encouraged to advocate for sustainable practices. Better patient outcomes because nurses who are more environmentally sensitive may be more aware of how environmental elements can affect a patient’s health and well-being.

This study focuses on understanding how the organizational context and culture can shape the impact of prosocial leadership behaviors on nurses’ development of a sustainability mindset. The adoption of environmentally responsible practices by nurses who are motivated by pro-social leadership and have a heightened understanding of sustainability is likely to contribute to the overall sustainability of the healthcare system. An organizational culture that values sustainability and pro-social leadership In the face of environmental difficulties, consciousness can improve healthcare organizations’ resilience and adaptation. Nurses can develop a sense of sustainability consciousness by pro-social leaders developing organizational ideals around sustainability, sharing their perspectives, and fostering mutual knowledge. Enhanced nurse well-being, job satisfaction, and engagement can result from pro-social leader behaviors that prioritize attending to nurses’ needs and fostering their professional development. By understanding the mechanisms that influence nurses’ sustainability consciousness, healthcare organizations can better support and empower nurses to become champions of sustainable healthcare.

The findings inform that programs for nursing education should include modules or courses that address sustainability consciousness, pro-social leadership practices, and how these affect individual, group, and organizational outcomes. Opportunities for experiential learning, such as case studies, role-plays, and simulations, should be made available to nursing students so they can acquire pro-social leadership techniques and comprehend how they might be used in actual healthcare settings. To serve as role models for aspiring nurses, nursing faculty should be prepared and taught to exhibit pro-social leadership behaviors in their mentorship and instruction.

In addition, healthcare organizations should create and put into effect activities that raise nurses’ awareness of sustainability as well as policies that reward and encourage pro-social leadership among nursing leaders. Programs for nurses’ professional growth and continuing education should cover pro-social leadership techniques, sustainability, and how to incorporate these into nursing practice. Pro-social leadership skills and a focus on sustainability should be included in the criteria and guidelines that nursing regulatory organizations and accreditation agencies develop for nursing practice and education. To guarantee a comprehensive strategy for fostering pro-social leadership and sustainability consciousness throughout the healthcare system, nursing policy should encourage multidisciplinary collaboration with other healthcare professions.

## Data Availability

The corresponding author can provide the datasets created and analyzed for this study upon reasonable request.
